# Heterogeneous BCR-ABL1 signal patterns identified by fluorescence in situ hybridization are associated with leukemic clonal evolution and poorer prognosis in BCR-ABL1 positive leukemia

**DOI:** 10.1186/s12885-019-6137-8

**Published:** 2019-10-08

**Authors:** Zhanglin Zhang, Zhiwei Chen, Mei Jiang, Shuyuan Liu, Yang Guo, Lagen Wan, Fei Li

**Affiliations:** 1 Department of Clinical Laboratory, the First Affiliated Hospital of Nanchang University, Nanchang, 330006 China; 2 Institute of Hematology, Academy of Clinical Medicine of Jiangxi Province, Nanchang, 330006 China; 3 Department of Hematology, the First Affiliated Hospital of Nanchang University, No. 17 Yongwai Street, Donghu District, Nanchang, 330006 Jiangxi China; 4 Jiangxi Key Laboratory of Molecular Diagnosis and Precision Medicine, Nanchang, 330006 China

**Keywords:** BCR-ABL1, Fluorescence in situ hybridization, Clonal evolution, Prognosis

## Abstract

**Background:**

Although extensive use of tyrosine kinase inhibitors has resulted in high and durable response rate and prolonged survival time in patients with BCR-ABL1 positive chronic myeloid leukemia (CML) and acute leukemia, relapse and drug resistance still remain big challenges for clinicians. Monitoring the expression of BCR-ABL1 fusion gene and identifying ABL kinase mutations are effective means to predict disease relapse and resistance. However, the prognostic impact of BCR-ABL1 signal patterns detected by fluorescence in situ hybridization (FISH) remains largely unaddressed.

**Methods:**

BCR-ABL1 signal patterns were analyzed using FISH in 243 CML-chronic phase (CML-CP), 17 CML-blast phase (CML-BP) and 52 BCR-ABL1 positive acute lymphoblastic leukemia (ALL) patients.

**Results:**

The patterns of BCR-ABL1 signals presented complexity and diversity. A total of 12 BCR-ABL1 signals were observed in this cohort, including 1R1G2F, 1R1G1F, 2R1G1F, 1R2G1F, 2R2G1F, 1R2G2F, 1R1G3F, 1G3F, 2G3F, 1G4F, 1R1G4F and 1R4F. Complex BCR-ABL1 signal patterns (≥ two types of signal patterns) were observed in 52.9% (*n* = 9) of the CML-BP patients, followed by 30.8% (*n* = 16) of the ALL patients and only 2.1% (*n* = 5) of the CML-CP patients. More importantly, five clonal evolution patterns related to disease progression and relapse were observed, and patients with complex BCR-ABL1 signal patterns had a poorer overall survival (OS) time compared with those with single patterns (5.0 vs.15.0 months, *p* = 0.006).

**Conclusions:**

Our data showed that complex BCR-ABL1 signal patterns were associated with leukemic clonal evolution and poorer prognosis in BCR-ABL1 positive leukemia. Monitoring BCR-ABL1 signal patterns might be an effective means to provide prognostic guidance and treatment choices for these patients.

## Background

BCR-ABL1 fusion gene, produced by the specific t (9;22) (q34;q11) chromosomal translocation, occurs in approximately 90% of the chronic myeloid leukemia (CML), 25% of the acute lymphoblastic leukemia (ALL) and less than 5% of the acute myeloid leukemia (AML) cases [1–3], and it constitutively encodes tyrosine kinase BCR-ABL1 oncoprotein, which is responsible for proliferative signals and leukemogenesis by activating Raf/MEK/ERK, PI3K/AKT, and JAK/STAT pathways [4, 5]. Although extensive use of tyrosine kinase inhibitors (TKIs) has resulted in high and durable response rate as well as prolonged survival time in BCR-ABL1 positive CML or ALL patients, relapse and drug resistance still remain big challenges for clinicians. Some studies have suggested that mutations in the BCR-ABL1 tyrosine kinase domain induce disease relapse or resistance to TKIs [6–8]. Moreover, the presence of +der(22) (9;22), *deletions* of the *derivative* chromosome *9* and other complex chromosome karyotypes are usually not sensitive to TKIs, suggesting worse clinical outcomes in these patients [9].

Conventional cytogenetic analysis (karyotyping) is the most commonly used method to confirm the presence of the t(9;22) and other chromosomal abnormalities [10, 11]. However, such analysis can not detect subtle changes, such as microdeletion. Meanwhile, monitoring the expression of BCR-ABL1 fusion gene by quantitative PCR (q-PCR) and identifying ABL kinase mutations by sequencing are also effective means to predict disease relapse and resistance. Fluorescence in situ hybridization (FISH) with locus-specific dual-color, dual-fusion probe (DCDF-FISH) not only confirms the presence of the t(9;22), but also shows typical or atypical signal patterns [12–14]. The atypical patterns usually represent deletions on the derivative chromosome 9 (−der 9 t(9;22)), three- or more-way variant t(9;22), gain of an additional Philadelphia chromosome (+der 22 t(9;22)) or other abnormalities [13]. However, the prognostic impact of BCR-ABL1 signal patterns identified by DCDF-FISH in BCR-ABL1 positive leukemia patients remains largely unaddressed.

In the present study, we reported the characteristics and evolution of BCR-ABL1signal patterns using FISH in 243 CML-chronic phase (CML-CP), 17 CML-blast phase (CML-BP) and 52 ALL patients. Our data indicated that monitoring BCR-ABL1 signal patterns might be an effective way to provide prognostic guidance and treatment choices for patients with BCR-ABL1 positive leukemia.

## Methods

### Patients

This study was performed in accordance with the guidelines of the Helsinki Declaration (1996) and approved by the Ethics Committee of the Institute of Hematology, the First Affiliated Hospital of Nanchang University. A total of 243 newly diagnosed CML-CP, 17 CML-BP and 52 newly diagnosed BCR-ABL1 positive ALL patients were enrolled in this study from March 2011 to June 2016. Written informed consents were obtained from all participants. All of the patients received TKI monotherapy (CML-CP patients) or TKI in combination with chemotherapy (CML-BP and ALL patients). Blood count, serum chemistry and BCR-ABL1 FISH were performed in all of the patients at the time of diagnosis. Subsequently, FISH for BCR-ABL1 was performed monthly in CML-BP and ALL patients, while not routinely checked later in CML-CP patients. Therefore, the clinical significance of BCR-ABL1 signal patterns was only evaluated in CML-BP and ALL patients. Table 1 lists the clinical parameters of CML-BP and ALL patients.

**Table 1:** FISH signal details in BCR-ABL1 positive leukemia patients

	CML-CP (*n* = 243)	CML-BP (*n* = 17)	BCR-ABL1+ ALL (*n* = 52)
FISH signals	1R1G1F;1R2G1F;2R1G1F; 2R2G1F;1R1G2F;1R1G3F	1R1G1F;1R1G2F;1R1G3F;1G4F; 1R2G1F;1R2G2F;1R2G3F;2R2G1F; 2R1G2F; 2R2G2F	1R1G1F;1R1G2F;1R1G3F; 1G4F;1R2G1F;1R2G2F;2R1G1F; 2R2G1F;1G3F;2G3F; 1R4F;1R1G4F
Complex signal patterns	1R1G2F/2R1G1F(n = 1)	1R1G1F/1R2G1F(n = 1)	1R1G2F/1R1G4F/1R1G3F (n = 1)
	1R1G2F/2R2G1F(n = 2)	1R2G1F/1R1G2F(n = 1)	1R1G2F/1R1G3F/1R2G2F (n = 1)
	1R1G2F/1R1G1F(*n* = 1)	1G4F/1R1G2F (n = 1)	1R1G2F/1R1G3F/1G3F/2R4F
	1R1G1F/2R1G1F(n = 1)	1R1G2F/1R2G2F/1R2G3F (n = 1)	(n = 1)
		2R1G2F/2R2G2F (n = 1)	1R1G3F/1R1G2F (*n* = 6)
		1R1G1F/1R2G2F (n = 1)	1R1G3F/1R1G2F/1G3F n = 1)
		1R1G2F/1R1G3F (*n* = 3)	1R2G2F/1RnG2F (n = 1)
			1R1G2F/1R1G4F (n = 1)
			2G3F/3F (n = 1) 1R1G2F/1G4F/2G8F (n = 1)
			1R1G2F/2R2G1F (n = 1)
			1R1G2F/1R2G1F (n = 1)
Typical single patterns	1R1G2F (n = 179)	1R1G2F (n = 5)	1R1G2F (n = 28)
Atypical single patterns	2R1G1F (n = 16)	1R1G1F (n = 1)	2R2G1F (n = 1)
	1R2G1F (n = 8)	1R1G3F(n = 2)	1R1G1F (n = 3)
	1R1G1F (*n* = 20)		1G3F (n = 1)
	2R2G1F (*n* = 14)		1R1G3F (n = 2)
	1R1G3F (n = 1)		2R1G1F (n = 1)

Some BCR-ABL1 signal patterns and their interpretations (R = red signal; G = green signal; F = fusion signal) [21]

1R1G2F: t(9;22)

1R2G1F: t(9;22) with deletion of the derivative chromosome 9 involving only the sequences 5′ of the ABL1 breakpoint

2R1G1F: t(9;22) with deletion of the derivative chromosome 9 involving only the chromosome 22 sequences 3′ of the BCR breakpoint

1R1G1F: t(9;22) with deletion of the derivative chromosome 9 involving sequences 5′ of the ABL1 breakpoint as well the chromosome 22 sequences 3′ of the BCR breakpoint

1R1G3F: t(9;22) with additonal Philadelphia chromosome

2R2G1F: Variant (three-or four-way) t(9;22)

1RnG2F: nG represents many added green signals that we are different to count

### Treatment response

Due to the small sample size of CML-BP patients, the treatment response was only evaluated in 52 newly diagnosed BCR-ABL1 positive ALL patients. Among these patients, only 3 patients received dasatinib in combination with chemotherapy, other 49 patients received imatinib based chemotherapies. Only two patients subsequently received allogeneic hematopoietic stem cell transplantation (allo-HSC). Complete remission (CR) and partial remission (PR) were determined based on morphological assessment of their bone marrow (BM) after a course of TKI in combination with chemotherapy. *CR* was *defined* by the *presence* of < *5*% *blasts* in the *BM*, with > *1* × *10*^*9*^/*L neutrophils* and > *100* × *10*^*9*^/*L platelets* in the peripheral blood with *no* detectable *extramedullary disease* (EMD). PR was defined by the presence of 5–19% blasts in the BM. *Relapse* was *defined* by *≥5*% *blasts* in the BM, circulating leukemic *blasts*, or the development of EMD.

### BCR-ABL1 fusion gene detected by DCDF-FISH

DCDF-FISH for BCR-ABL1 (Vysis Inc., Downers Grove, IL, USA) was performed on BM cells prepared according to the routine FISH methods. At least 200 cells with well-delineated signals were evaluated. The cut-off level was set as 0.98% according to mean ± standard deviation in twenty normal controls.

### Statistical analysis

Statistical analysis was conducted using SPSS software (version 22.0). Categorical variables were compared using nonparametric tests. Overall survival (OS) was calculated from *date* of initial *diagnosis until death*. Survival curves were plotted by the Kaplan–Meier method, and statistical differences between the curves were analyzed using the log-rank test. Multivariate analysis of variables associated with survival was conducted by Cox Proportional-Hazard model for OS. *P* ≤ 0.05 was considered as statistically significant.

## Results

### The patterns of BCR-ABL1 signal present complexity and diversity

**Fig. 1:**
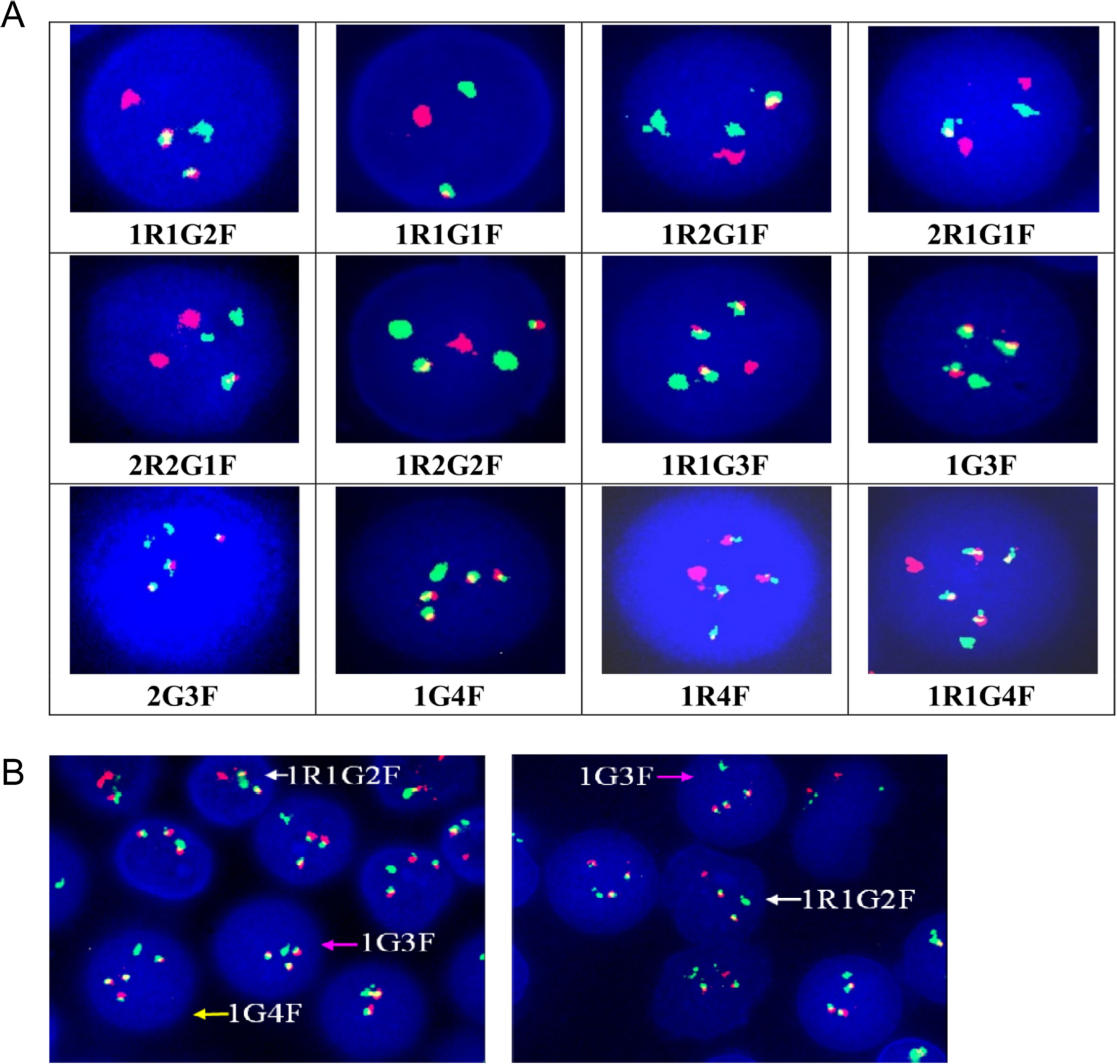
The patterns of BCR-ABL1 signals presented as complexity and diversity detected by specific dual-color, dual-fusion FISH probe (DCDF-FISH). (**a**) Twelve types of BCR-ABL1 signals were observed in CML-CP, CML-BP and ALL patients. (**b**) Complex BCR-ABL1 signal patterns (two or three BCR-ABL1 signals) could be observed in the same patient

To explore the characteristics of BCR-ABL1 signals among CML-CP, CML-BP and ALL patients, we assessed and analyzed BCR-ABL1 signals in 243 CML-CP, 17 CML-BP and 52 BCR-ABL1 positive ALL patients using DCDF-FISH. The classic BCR-ABL1 FISH pattern has two fusions, each fusion includes one ABL signal and one BCR signal. However, we found that the BCR-ABL1 signal patterns presented complexity and diversity in this cohort (Table 1). We observed a total of 12 types of BCR-ABL1 signals, including 1R1G2F, 1R1G1F, 2R1G1F, 1R2G1F, 2R2G1F, 1R2G2F, 1R1G3F, 1G3F, 2G3F, 1G4F, 1R1G4F and 1R4F (Fig. 1a and Table 1). Interestingly, some patients presented two or more BCR-ABL1 signals simultaneously (Fig. 1b).

### Complex BCR-ABL1 signal patterns are more frequently detected in CML-BP and ALL patients

**Fig. 2:**
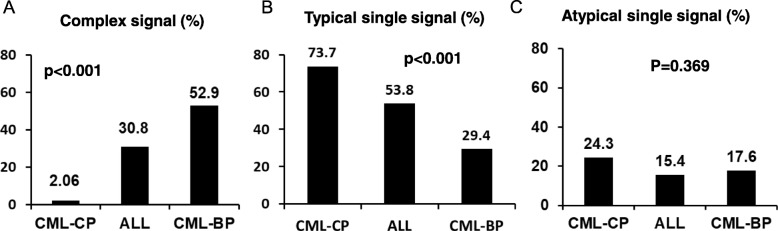
Complex BCR-ABL1 signal patterns were more frequently detected in CML-BP and ALL patients. (**a**) Complex BCR-ABL1 signal patterns were observed in 52.9% of the CML-BP patients, followed by 30.8% of the ALL patients and only 2.1% of the CML-CP patients (*p* < 0.001). (**b**) Typical single BCR-ABL1 signal pattern was observed in 29.4% of the CML-BP, 53.8% of the ALL patients and 73.7% of the CML-CP patients (*p* < 0.001). (C) The proportions of patients with atypical single BCR-ABL1 signal patterns were similar among three groups (17.6, 15.4 and 24.3%) (*p* = 0.369)

We further found that only six types of signals were observed in CML-CP patients, while 12 and 10 types of signals were detected in ALL and CML-BP patients, respectively (Table 1). Next, we indentified two or more BCR-ABL1 signal patterns as complex BCR-ABL1 signal patterns. Typical single BCR-ABL1 signal pattern means single 1R1G2F fusion signal. Atypical single BCR-ABL1 signal pattern indicates single BCR-ABL1 fusion signals other than 1R1G2F (such as 1R1G1F or 1R1G3F). Our results showed that complex BCR-ABL1 signal patterns were observed in 52.9% (*n* = 9) of the CML-BP patients, followed by 30.8% (*n* = 16) of the BCR-ABL1 positive ALL patients and only 2.1% (*n* = 5) of the CML-CP patients (*p* < 0.001) (Fig. 2a). Conversely, typical single BCR-ABL1 signal pattern was observed in 29.4% (*n* = 5) of the CML-BP patients, 53.8% (*n* = 28) of the BCR-ABL1 positive ALL patients and 73.7% (*n* = 179) of the CML-CP patients (*p* < 0.001) (Fig. 2b). The proportions of patients with atypical BCR-ABL1 signal patterns were similar, accounting for 17.6% (*n* = 3), 15.4% (*n* = 8) and 24.3% (*n* = 59) in the CML-BP, ALL and CML-CP patients, respectively (*p* = 0.369) (Fig. 2c). The expressed patterns of BCR-ABL1 signal were significantly different among the three groups (*p* < 0.001). These data suggested that ALL and CML-BP patients possessed more heterogeneous BCR-ABL1 cloned cells, indicating greater chromosomal abnormality and genomic instability. Due to the limited space of article, we listed the FISH signal details in BCR-ABL1 positive ALL patients in the Additional file 1: Table S1.

### The comparison of clinical features between BCR-ABL1 positive CML-BP and ALL patients

We compared whether there was any difference in clinical features between BCR-ABL1 positive ALL and CML-BP patients. We analyzed the clinical data including age, sex, leukocyte count, hemoglobin count, thrombocyte count, cytogenetic abnormality, BCR-ABL1 FISH signal pattern and splenomegaly, in 17 CML-BP patients and 52 ALL patients. Except that moderate-severe splenomegaly was more often found in the CML-BP patients (*p* < 0.001) and lower thrombocyte count was more often detected in the BCR-ABL1 positive ALL patients (*p* < 0.001), there was no difference between the two groups (Table 2). We further compared the clinical features in BCR-ABL1 positive ALL patients with complex and single signal patterns, but the difference was not statistically significant (Table 3).

**Table 2:** The clinical features of CML-BP and BCR-ABL1 positive ALL patients

Characteristics	CML-BP	BCR-ABL1^+^ ALL	*P* value
Number of patients	17	52	
Median Age, y (Range)	42.9 (15–70)	39.0 (13–76)	0.306
Male/Female, %	52.9/47.1	57.7/42.3	0.631
Leukocyte count, 10^9^/L (range)	97.2 (1.0–303)	57.4 (1.1–308)	0.131
Hemoglobin, g/L (range)	82.5 (48–132)	87.3 (38–149)	0.630
Thrombocyte count, 10^9^/L (range)	268.5 (13–2488)	43.0 (3–230)	0.000
LDH, U/L (range)	1059 (98–5490)	836 (156–5353)	0.526
Cytogenetic abnormalities, %			0.684
t (9;22)	4/17 (23.5%)	11/52 (21.2%)	
ACAs	5/17 (29.4%)	11/52 (21.2%)	
No split phase	8/17 (47.1%)	30/52 (57.7%)	
BCR-ABL1 FISH			0.068
Complex signal pattern	9/17 (52.9%)	16/52 (30.8%)	
Typical single pattern	5/17 (29.4%)	28/52 (53.8%)	
Atypical single pattern	3/17 (17.6%)	8/52 (15.4%)	
Splenomegaly			0.000
Normal-mild	8/17 (47.1%)	51/52 (98.0%)	
Moderate- severe	9/17 (52.9%)	1/52 (2.0%)	

Abbreviations: *CML-BP* = chronic myeloid leukemia-blast phase, *ALL* = acute lymphoblastic leukemia, *LDH* = Lactate dehydrogenase, *ACAs* = additional cytogenetic abnormalities, *Complex signal pattern* = two or more types of BCR-ABL signal patterns, *Single pattern* = single 1R1G2F fusion signal or other single BCR-ABL fusion signals other than 1R1G2F. Mild splenomegaly: <3 cm under the ribs; Moderate splenomegaly: 3 ~ 6 cm under the ribs; Severe splenomegaly: >6 cm under the ribs

**Table 3:** Comparison of patients’characteristics at diagnosis in BCR-ABL1 positive ALL with complex and single patterns

Characteristics	Complex patterns	Single pattern	*P* value
Number of patients	16	36	
Median Age, y (Range)	38.3 (16–61)	39.0 (13–76)	0.945
Male/Female, %	43.8/56.3	57.7/42.3	0.124
Leukocyte count, 10^9^/L (range)	41.4 (1.2–106)	57.4 (1.1–308)	0.753
Hemoglobin, g/L (range)	84.9 (38–127)	87.3 (38–149)	0.784
Thrombocyte count, 10^9^/L (range)	47.8 (7–230)	43.0 (3–230)	0.753
LDH, U/L (range)	978 (250–5353)	766 (156–3723)	0.364
Cytogenetic abnormalities, %			0.379
t (9;22)	2/16 (12.5%)	9/36 (25.0%)	
ACAs	5/16 (31.3%)	6/36 (16.7%)	
No split phase	9/16 (56.3%)	21/36 (58.3%)	

### BCR-ABL1 clonal evolution in ALL patients predicts disease progression and relapse

**Fig. 3:**
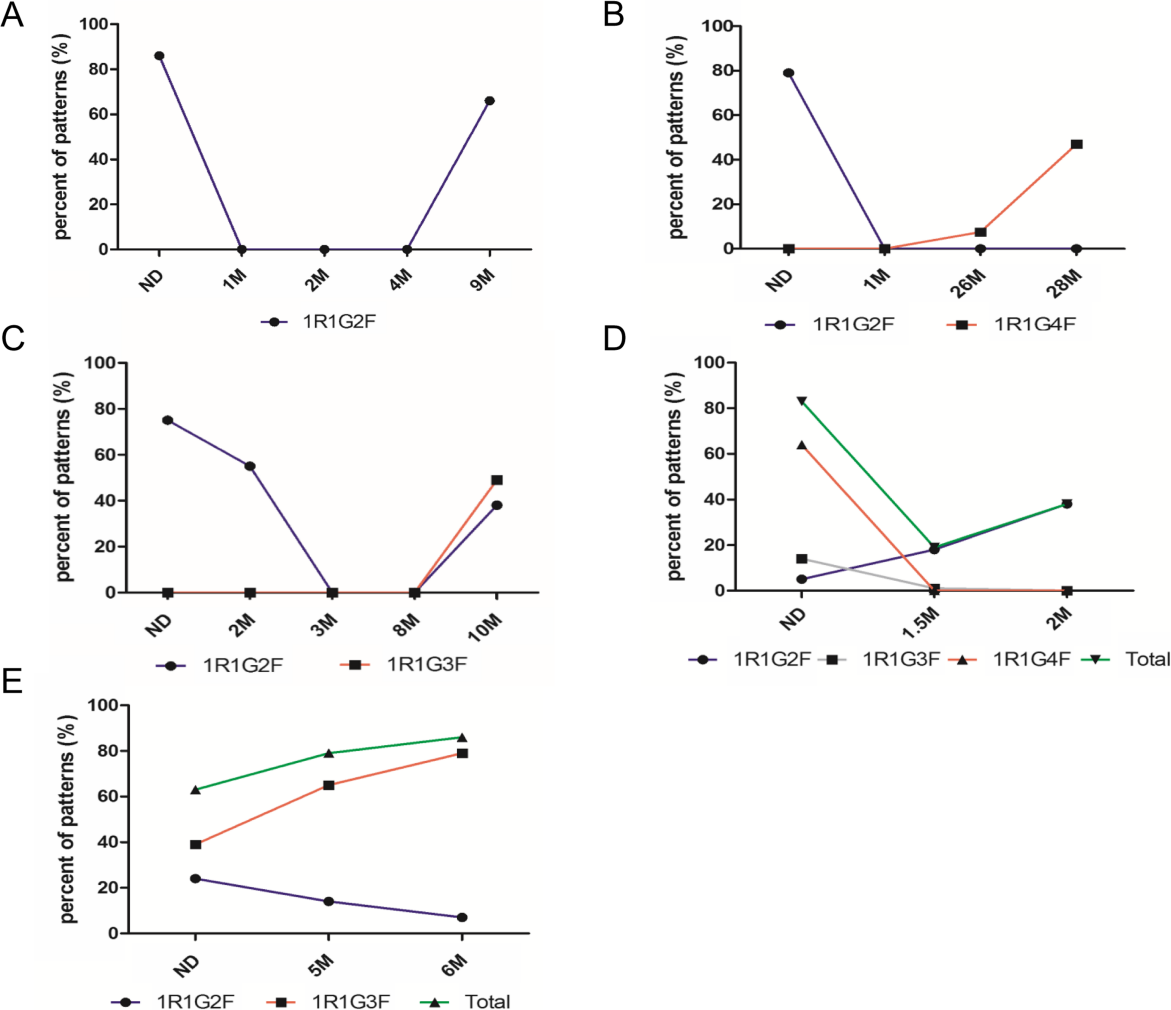
BCR-ABL1 clonal evolution in ALL patients predicted disease progression and relapse. (**a**) 1R1G2F was sensitive single clone at disease onset, which disappeared after treatment, and it was still the primary clone (1R1G2F) during relapse. (**b**) 1R1G2F was sensitive single clone at disease onset, which disappeared after treatment, whereas new single clone (1R1G4F) was observed during relapse. (**c**) 1R1G2F was sensitive single clone at disease onset, whereas new and primary clones (1R1G2F and 1R1G3F) simultaneously occurred during relapse. (**d**) 1R1G2F, 1R1G3F and 1R1G4F presented different subclones during disease onset, some subclones (1R1G4F and 1R1G3F) were sensitive, whereas minor subclones (1R1G2F) were resistant. (**d**) 1R1G2F and 1R1G3F presented two different subclones at disease onset. Minor subclones (1R1G2F) were sensitive, whereas the preponderant subclones (1R1G3F) were resistant

More importantly, we further found that the development of BCR-ABL1 signal patterns could indicate leukemic clonal evolution. Disease progression and relapse can be predicted by sequentially monitoring the BCR-ABL1 modes at different time points using FISH. In the present study, we observed that five clonal evolution modes were related to disease progression in BCR-ABL1 positive ALL patients. For example, clonal evolution modes in five patients were respectively listed below (Fig. 3). Patient one presented sensitive single clone (1R1G2F) at disease onset, which disappeared after treatment, and it was still observed as the primary clone (1R1G2F) during relapse (Fig. 3a). Patient two presented sensitive single clone (1R1G2F) at disease onset, which disappeared after treatment, whereas new single clone (1R1G4F) was observed during relapse (Fig. 3b). Patient three presented sensitive single clone (1R1G2F) at disease onset, whereas new and primary clones (1R1G2F and 1R1G3F) simultaneously occurred during relapse (Fig. 3c). The fourth patient presented many different subclones (1R1G2F, 1R1G3F and 1R1G4F) during disease onset, some sensitive subclones (1R1G4F and 1R1G3F) disappeared after treatment, whereas minor resistant subclones (1R1G2F) gradually progressed to preponderant subclones until relapse (Fig. 3d). The fifth patient simultaneously presented two different subclones (1R1G2F and 1R1G3F) at disease onset. Minor subclones (1R1G2F) were sensitive and decreased after treatment, whereas the preponderant subclones (1R1G3F) were resistant to TKIs or chemotherapy drugs (Fig. 3e). Regrettably, due to the retrospective analysis and the incomplete data, we did not provide the accurate incidence of different modes in all ALL patients.

### Complex BCR-ABL1 signal patterns are associated with a poorer survival compared with single pattern in ALL patients

**Fig. 4:**
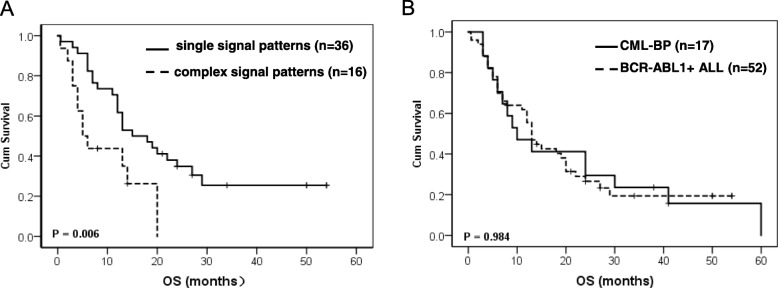
The analysis of survival in BCR-ABL1 positive ALL and CML-BP patients. (**a**) BCR-ABL1 positive ALL patients with complex signal patterns had poor OS time compared with patients with single signal patterns (*P* = 0.006). (**b**) BCR-ABL1 positive ALL and CML-BP patients had similar OS time (*P* = 0.984)

According to above-mentioned findings, complex BCR-ABL1 signal patterns were more frequently found in ALL and CML-BP patients, which could predict genomic instability. We further analyzed the prognostic factors for OS time in this cohort. The median follow-up time was 13.0 months (range, 1.0–54.0 months). Figure 4a and Table 4 reveal that patients with BCR-ABL1 complex signal patterns (5.0 vs. 15.0 months, *p* = 0.006), additional cytogenetic abnormalities (ACAs) (6.0 vs. 27.0 months, *p* = 0.001) or without achieving CR + PR (7.0 vs. 19.0 months, *p* = 0.019) had a poorer OS time compared with control patients. Meanwhile, in thirty patients with no split phase, patients with complex BCR-ABL1 pattern (*n* = 9) have poorer OS time than patients with single BCR-ABL1 pattern (n = 9) (9.6 vs 28.3 months, *p* = 0.026). However, due to the limited number of patients, multivariate analysis showed that only ACA was the independent prognostic factor for OS (HR: 0.16, 95% CI: 0.05–0.55, *p* = 0.004) (Table 5).

**Table 4:** Univariate analysis of risk factors for OS in BCR-ABL1 positive ALL

Group	Numbers	OS (range, month)	*P* value
Leukocyte count, 10^9^/L			0.736
≥ 50	21	13 (6.5~19.5)	
< 50	29	14 (6.7~21.3)	
LDH			0.775
≥ 250 U/L	37	13.0 (10.7~15.3)	
< 250 U/L	10	8.0 (0.0~27.3)	
CR + PR			0.019
Yes	44	19.0 (12.7~25.2)	
No	5	7.0 (4.4~9.6)	
BCR-ABL1 FISH			0.006
Complex signal patterns	36	5.0 (2.4~7.6)	
single patterns	16	15.0 (8.3~21.7)	
Cytogenetic abnormalities			0.001
t (9;22)	11	27 (14.5~39.5)	
ACAs	11	6 (1.7~10.3)	

Abbreviations: *LDH* lactic dehydrogenase, *CR* complete remission, *PR* partial remission

**Table 5:** Multivariate analysis of risk factors for OS in BCR-ABL1 positive ALL

Prognostic parameters (yes)	HR for OS (95% CI)	*P* value
CR + PR	0.45 (0.13–1.54)	0.201
Complex BCR-ABL1 signal patterns	0.44 (0.14–1.35)	0.152
ACAs	0.16 (0.05–0.55)	0.004

## Discussion

Traditional drug resistance in BCR-ABL1 positive patients caused by mutations in the tyrosine kinase domain of BCR-ABL1 or quiescent leukemic stem cells sheltered in unexposed region of BM has been widely accepted [6, 8]. Disease relapse and resistance restrain the clinical outcomes of CML and ALL patients, urging us to explore pathogenesis of leukemia. ACAs caused by genomic instability in leukemic cells are inevitable in progression of leukemia, and its prognostic significance in the setting of TKIs remains largely unexplored. Even under the TKI treatments (imatinib monotherapy or imatinib in combination with low-dose cytarabine or interferon), the frequency of these ACAs in CML increases the probability from chronic phase to blast phase and confers poor survival, which has been proved in a randomized CML study IV with 1151 cases [15]. Several gene mutations or other fusion genes, such as P53, RB, GATA-2 and AML1-EVI-1 fusion genes, cause the fatal blast crisis and lead to a shorter OS time in CML patients [11, 16–18]. Conversely, though some studies have reported that there is a significantly higher rate of complete cytogenetic response (CCyR) at 6 months (*p* = 0.02) for CML patients without ACAs, the cumulative CCyR and major molecular response (MMR) rates are not different between patients with and without ACAs. Similarly, MR4.0 and MR4.5 rates are similar in both groups, indicating that ACAs at diagnosis do not significantly impact transformation-free survival, failure-free survival, event-free survival or OS in CML-CP patients [19].

Recently, Nicholas J. Short et al. [9] have shown that +der(22)t(9;22) and/or − 9/9p in the absence of high hyperdiploidy are independent factors for worse relapse-free *survival* (RFS) (HR 2.03 [95% CI 1.08–3.30], *p* = 0.03) and OS (HR 2.02 [95% CI 1.10–3.71], *p* = 0.02) in Philadelphia+ ALL patients receiving chemotherapy in combination with a TKI treatment (imatinib or dasatinib). To date, monitoring the expression of BCR-ABL1 fusion gene by q-PCR and identifying ABL kinase mutations by sequencing have been employed as effective means to predict disease relapse and resistance in CML and ALL patients. However, these technologies can not detect ACAs in patients. At diagnosis, the presence of clonal ACAs may be observed in 5% of CML-CP patients, ~ 30% of AP patients and ~ 80% of patients with blast crisis [20]. Conventional karyotyping analysis can identify some obvious ACAs but not subtle changes. DCDF-FISH not only confirms the presence of t(9;22), but also identifies deletions on the derivative chromosome 9 (−der 9 t(9;22)), three- or more-way variant t(9;22), gain of an additional Philadelphia chromosome (+der 22 t(9;22)) or other abnormalities [13]. Jain et al. [21] have analyzed 1076 CML patients with positive BCR-ABL1 using a commercially available BCR-ABL1 dual-color, dual-fusion probe. Typical dual-fusion signals are seen in 74% of cases. Atypical signal patterns are seen in 26% of cases. 1F1R2G (4%), 1F2R1G (2.5%) and 1F1R1G (11%) represent derivative deletions in chromosome 9 sequence, chromosome 22 sequence, or both, respectively. 3F1R1G (6.5%) usually represents gain of an additional Philadelphia chromosome; and 1F2R2G (1%) represents a three- or four-way variant translocation. More than one signal pattern are seen in 1% of cases. In the present study, our results indicated that complex BCR-ABL1 signal patterns were more frequently found in CML-BP (52.9%) and BCR-ABL1 positive ALL (30.8%) patients, while they were rarely detected in CML-CP (2.1%) patients. There were only six types of signals observed in CML-CP patients, while 12 and 10 types of signals were found in ALL and CML-BP patients, respectively, suggesting that ALL and CML-BP patients possessed more heterogeneous BCR-ABL1 cloned cells and ACAs.

Tumor heterogeneity originates from multiple genetic and epigenetic diversities, leading to clonal evolution and drug resistance. CML-BP patients with simultaneous ACAs show lower response rates and a shorter failure time of imatinib mesylate (STI571) treatment [22]. BCR-ABL1 independent gene mutations (33% of patients had somatic mutations in addition to BCR-ABL1, including ASXL1, DNMT3A, RUNX1 and TET2, revealing that most mutations were part of the Ph-positive clones) are frequently found in Ph-negative and Ph-positive clones of CML patients and may be considered as important cofactors in the clonal evolution of CML [23]. Moreover, BCR-ABL1 compound mutations and other ABL1 tyrosine kinase inhibitors (TKIs) can also confer high-level resistance to imatinib [24]. Several research groups have also screened relapse-related gene mutations, including RAS and CREBBP/NT5C2 mutations in ALL patients [25, 26]. However, the correlation between clonal evolution and FISH signal patterns has not been well established in BCR-ABL1 positive patients. Therefore, we monitored the evolution of FISH signal patterns in BCR-ABL1 positive ALL patients. A total of five clonal evolution patterns related to disease progression were observed, and various sensitive or drug resistant subclones were found in patients receiving TKI treatment and chemotherapy. Therefore, we believed that monitoring the BCR-ABL1 signal patterns using FISH could also be a effective way to predict the disease progression and relapse for BCR-ABL1 positive ALL patients. Regrettably, we only evaluated 200 cells which might be missed some small clones. Moreover, due to the incomplete data and retrospective analysis, there is maybe not just five modes involved clone evolution. In the future, we will prospectively explain its incidence and clinical significance in the larger size of patients. Because we did not monthly check the BCR-ABL1 using FISH in CML-CP patients subsequently and due to the small sample size of CML-BP patients, we only evaluated the treatment response in BCR-ABL1 positive ALL patients and survival time in CML-BP and ALL patients. Our results indicated that patients with complex BCR-ABL1 signal patterns, ACAs and without achieving CR + PR had a poor OS time. In addition, among 30 ALL patients with no split phase, nine patients with complex BCR-ABL1 pattern had poorer OS time than patients with single BCR-ABL1 pattern (9.6 vs 28.3 months, *p* = 0.026). So, we think ACAs by karyotyping and FISH by DCDF probes could be well used complimentary to select more poor-risk patients. FISH analysis would be specially helpful for those patients with no split phase. Conversely, ACAs might identify poor-risk patients within the group of single-pattern patients. Due to the limited number of patients, we only observed ACAs was the independent prognostic factor for OS. Furthermore, our data also indicated CML-CP patients once progressed to blast phase, had similarly poor survival with BCR-ABL1 positive ALL patients (median OS is 10.0 vs.13.0 months, *p* = 0.984) even under the background of TKI treatment (Fig. 4b). Receiving hematopoietic stem cell transplantation or next generation of TKI as soon as possible might overcome the poor prognostic effect.

## Conclusions

Taken together, our results suggested that signal patterns of BCR-ABL1 identified by FISH could predict disease progression and OS in BCR-ABL1 positive acute leukemia. Of course, our study had some limitations, such as the small sample size of CML-BP patients. Moreover, we did not analyze the correlation among the results of BCR-ABL1 FISH, q-PCR and sequencing which might be related to relapse or resistance to TKI-based therapy [26]. These questions need to be further answered in larger number of patients.

## Supplementary information

**Additional file 1: Table S1.** FISH signal details in BCR-ABL1 positive ALL patients.

## Data Availability

The datasets supporting the conclusions of this manuscript are included within the article. Please contact author for raw data requests.

## Abbreviations

ACAs: Additional cytogenetic abnormalities
ALL: Acute lymphoblastic leukemia
AML: Acute myeloid leukemia
CCyR: Complete cytogenetic response
CML-BP: Chronic myeloid leukemia-blast phase
CR: Complete remission
DCDF: Dual color, dual fusion probe
FISH: Fluorescence in situ hybridization
LDH: Lactate dehydrogenase
MMR: Major molecular response
OS: Overall survival
PR: Partial remission
TKIs: Tyrosine kinase inhibitors
