# Supplementary material for: Heterogeneous BCR-ABL1 signal patterns identified by fluorescence in situ hybridization are associated with leukemic clonal evolution and poorer prognosis in BCR-ABL1 positive leukemia
Source: BMC Cancer. 2019 Oct 8;19:935. doi: 10.1186/s12885-019-6137-8 (PMC6781398; doi:10.1186/s12885-019-6137-8)
Supplement: Supplementary file 1 — Additional file 1: Table S1. FISH signal details in BCR-ABL1 positive ALL patients. [file 12885_2019_6137_MOESM1_ESM.docx]

**Supplementary table 1. FISH signal details in BCR-ABL1 positive** **ALL patients.**

| **Number** | | **FISH** | **Karyotype** |  |  |  |  |  |  |  |
| --- | --- | --- | --- | --- | --- | --- | --- | --- | --- | --- |
| **complex signal patterns （n=16）** | | |  | | | | |  |  |  |
| 1 | 5%1R1G2F/64%1R1G4F/18%1R1G3F | | 46,XY,i (8)(q10), iuer(9) t(9;22) (q34;q11)(23)/47,idem(22)t(9;22) (7) | | | | |  |  |  |
| 2 | 35%1R1G2F/30%1R1G3F/15%1R2G2F | | 47,XX,+9,t(9;22)(q34;q11)*2(22)/48-46,XX,+9,(22)t(9;22)*2,+11...(13)/46,XX (2) | | | | |  |  |  |
| 3 | 80%1R1G2F/1R1G3F(n)/1G3F/2R4F(n) | | N | | | | |  |  |  |
| 4 | 74%1R1G3F/15%1R1G2F | | N | | | | |  |  |  |
| 5 | 28%1R1G3F/24%1R1G2F | | N | | | | |  |  |  |
| 6 | 80%1R1G3F/1R1G2F(n)/1G3F(n) | | N | | | | |  |  |  |
| 7 | 86%1R1G2F/4%1R1G3F | | N | | | | |  |  |  |
| 8 | 80%1R1G2F/60%1R1G3F | | 46,XY,t(9;22)(q34;q11)(20) | | | | |  |  |  |
| 9 | 90%1R1G2F/2%1R1G3F | | 44-46,XY,t(9;22) (q34;q11)(7);49-50,XX,+9,t(9;22);+19,ider(22)t(9;22)(2);46,XY(3) | | | | |  |  |  |
| 10 | 3%1R2G2F/4%1RnG2F | | N | | | | |  |  |  |
| 11 | 4%1R1G2F/81%1R4GF | | N | | | | |  |  |  |
| 12 | 48%2G3F/16%3F | | N | | | | |  |  |  |
| 13 | 3%1R1G2F/86%1G4F/4%2G8F | | 49-52,XX,+5,+8,t(9;22) (q34;q11),+der(22)t(9;22)..... (20) | | | | |  |  |  |
| 14 | 38%1R1G2F/45%2R2G1F | | 46,XY,add(2)(p21),t(9;22)(q34;q11)del(9)(p22)(2)/46,XY(18) | | | | |  |  |  |
| 15 | 15%1R1G3F/70%1R1G2F | | N | | | | |  |  |  |
| 16 | 34%1R1G2F/23%1R2G1F | | 46,XY,t(9;22)(q34;q11)(20) | | | | |  |  |  |
| **Typical-single fusion pattern （n=28）** | | |  | | | | |  |  |  |
| 17 | 89%1R1G2F | | 45,XY,-7,t(9;22)(q34;q11)(5)/46,XY(3) | | | | |  |  |  |
| 18 | 49%1R1G2F | | 48,XY,+X.+2,t(9;22)(q34;q11)(13)/46,XY(7) |  |  |  |  |  |  |  |
| 19 | 8%1R1G2F | | 46,XY,t(9;22)(q34;q11)(20) |  |  |  |  |  |  |  |
| 20 | 65%1R1G2F | | 46,XY,t(9;22)(q34;q11)(20) |  |  |  |  |  |  |  |
| 21 | 47%1R1G2F | | N |  |  |  |  |  |  |  |
| 22 | 92%1R1G2F | | N |  |  |  |  |  |  |  |
| 23 | 72%1R1G2F | | 43-45,X,-X,t(9;22)(q34;q11)...(8)/46,XX(7) |  |  |  |  |  |  |  |
| 24 | 80%1R1G2F | | N |  |  |  |  |  |  |  |
| 25 | 68%1R1G2F | | N |  |  |  |  |  |  |  |
| 26 | 49%1R1G2F | | 44-46,XY,t(9;22)(q34;q11)......(12) |  |  |  |  |  |  |  |
| 27 | 80%1R1G2F | | 46,XY,t(9;22)(q34;q11)(15) |  |  |  |  |  |  |  |
| 28 | 80%1R1G2F | | N |  |  |  |  |  |  |  |
| 29 | 64%1R1G2F | | N |  |  |  |  |  |  |  |
| 30 | 70%1R1G2F | | N |  |  |  |  |  |  |  |
| 31 | 72%1R1G2F | | N |  |  |  |  |  |  |  |
| 32 | 69%1R1G2F | | N |  |  |  |  |  |  |  |
| 33 | 79%1R1G2F | | N |  |  |  |  |  |  |  |
| 34 | 70%1R1G2F | | 46,XX,add(8)(q24),t(9;22)(q34;q11)(12)/46,XY(2) | |  |  |  |  |  |  |
| 35 | 81%1R1G2F | | 46,XY,t(9;22) (q34;q11)(11) |  |  |  |  |  |  |  |
| 36 | 75%1R1G2F | | N |  |  |  |  |  |  |  |
| 37 | 88%1R1G2F | | 46,XY,t(9;22)(q34;q11)(2)/46,XY(1) |  |  |  |  |  |  |  |
| 38 | 89%1R1G2F | | N |  |  |  |  |  |  |  |
| 39 | 12%1R1G2F | | 46,XY,t(9;22)(q34;q11)(2)/46,XY(11) |  |  |  |  |  |  |  |
| 40 | 89%1R1G2F | | N |  |  |  |  |  |  |  |
| 41 | 86%1R1G2F | | N |  |  |  |  |  |  |  |
| 41 | 24.5%1R1G2F | | / |  |  |  |  |  |  |  |
| 43 | 89%1R1G2F | | 44-46,XY,t(9;22)(q34;q11)(13)/46,XY(2) |  |  |  |  |  |  |  |
| 44 | 38%1R1G2F | | N |  |  |  |  |  |  |  |
| **Atypical-single fusion pattern （n=8）** | | |  |  |  |  |  |  |  |  |
| 45 | 86%2R2G1F | | N |  |  |  |  |  |  |  |
| 46 | 71%1R1G1F | | N |  |  |  |  |  |  |  |
| 47 | 76%1R1G1F | | N |  |  |  |  |  |  |  |
| 48 | 72%1R1G1F | | 46,XY,+19q,t(9;22)(q34;q11)(1)/46,XY(19) |  |  |  |  |  |  |  |
| 49 | 80%1G3F | | N |  |  |  |  |  |  |  |
| 50 | 74%1R1G3F | | N |  |  |  |  |  |  |  |
| 51 | 74%1R1G3F | | N |  |  |  |  |  |  |  |
| 52 | 64%2R1G1F | | 45,XY,-7,t(9;22)(q34;q11)(10) |  |  |  |  |  |  |  |

N: no split phase; n: positive (above 0.98%) but the detailed percentage was not available
